# Comprehensive Antigen Screening Identifies *Moraxella catarrhalis* Proteins That Induce Protection in a Mouse Pulmonary Clearance Model

**DOI:** 10.1371/journal.pone.0064422

**Published:** 2013-05-09

**Authors:** Margarita Smidt, Patrick Bättig, Suzanne J. C. Verhaegh, Axel Niebisch, Markus Hanner, Sanja Selak, Wolfgang Schüler, Eva Morfeldt, Christel Hellberg, Eszter Nagy, Urban Lundberg, John P. Hays, Andreas Meinke, Birgitta Henriques-Normark

**Affiliations:** 1 Intercell AG, Campus Vienna Biocenter 3, Vienna, Austria; 2 Department of Microbiology, Tumor and Cell Biology, Karolinska Institutet, Stockholm, Sweden; 3 Department of Medical Microbiology and Infectious Diseases, Erasmus MC, Rotterdam, The Netherlands; Instituto Butantan, Brazil

## Abstract

*Moraxella catarrhalis* is one of the three most common causative bacterial pathogens of otitis media, however no effective vaccine against *M. catarrhalis* has been developed so far. To identify *M. catarrhalis* vaccine candidate antigens, we used carefully selected sera from children with otitis media and healthy individuals to screen small-fragment genomic libraries that are expressed to display frame-selected peptides on a bacterial cell surface. This ANTIGENome technology led to the identification of 214 antigens, 23 of which were selected by *in vitro* or *in vivo* studies for additional characterization. Eight of the 23 candidates were tested in a *Moraxella* mouse pulmonary clearance model, and 3 of these antigens induced significantly faster bacterial clearance compared to adjuvant or to the previously characterized antigen OmpCD. The most significant protection data were obtained with the antigen MCR_1416 (Msp22), which was further investigated for its biological function by *in vitro* studies suggesting that Msp22 is a heme binding protein. This study comprises one of the most exhaustive studies to identify potential vaccine candidate antigens against the bacterial pathogen *M. catarrhali*s.

## Introduction

*Moraxella catarrhalis* is a gram-negative aerobic diplococcus and an exclusive human respiratory pathogen that for a long time used to be considered a purely human commensal [1]. However, *M. catarrhalis* is the third most frequent bacterial pathogen causing otitis media disease in children (after *Streptococcus pneumoniae* and non-typeable *Haemophilus influenzae* (NTHI)), and is a major cause of exacerbations in adults with chronic obstructive pulmonary disease (COPD) [2]. Further, between 50–85% of all children experience at least one acute otitis media (AOM) episode before 3 years of age [3], [4], and the disease is associated with high costs. In addition, chronic and frequent recurrent AOM can lead to delayed speech development and language learning, due to hearing impairment. Moreover, complications including mastoiditis, and in rare cases even meningitis, may develop as a result of such middle ear infections [5], [6].

Since otitis media (OM) is a polymicrobial disease, an effective vaccine will have to protect against the 3 main bacterial causative agents of OM, including *M. catarrhalis,* and several vaccine related studies have already been performed to identify potential single vaccine candidates. These include various outer membrane proteins (OMPs) and lipooligosaccharide [7], [8]. Others have used a genome-wide data mining approach to identify novel antigens [9]. Of the putative antigens so far identified, the ubiquitous surface proteins A (UspA1, UspA2, and UspA2H) [10], [11], involved in adherence [12] and serum resistance [13], have been shown to provide some protection in animal models using active vaccination or passive immunization strategies. Other potential candidates include the IgD-binding protein Hag/MID, a human epithelial cell adhesin and B cell mitogen, [14], and it has been reported that a monoclonal antibody specific for the outer membrane protein CopB, an iron-regulated protein involved in iron uptake from transferrin and lactoferrin, enhanced pulmonary clearance of *M. catarrhalis* in a mouse model [15]. Finally, the porin OmpCD, an adhesin, was reported to enhance pulmonary clearance upon immunization [16], and at the time that this research project began, appeared to be the most appropriate potential vaccine candidate to act as a positive control in *in vivo* immunization experiments.

The ANTIGENome technology offers another approach in the search for vaccine candidates and has been successfully applied to identify novel protective antigens from several other bacterial pathogens [17], [18], [19]. The technology generates many thousands of potential peptide antigen candidates that are then screened using magnetic-activated cell sorting (MACS) methods against well characterized human sera to identify novel protein vaccine candidates.

We have applied this technology and selected 214 protein candidates, among them the previously described protective proteins, UspA, Hag, CopB and OmpCD. Further validation by *in vitro* assays and finally in a murine model of *Moraxella* pulmonary clearance identified three proteins from *M. catarrhalis* as novel protective vaccine candidates. The functional characterization of one of these candidates, the surface protein Msp22, in *Moraxella* showed that it possesses heme-dependent peroxidase activity.

## Materials and Methods

### Ethical statement

All human serum samples used for these studies were collected according to the general national ethical guidelines and upon consent from individual subjects. Sera from healthy individuals were collected for this and similar studies by Intercell with written consent given by each individual specifically for this study. Collection of sera at the Erasmus University Medical Center (Rotterdam) was approved specifically for this study by the medical ethical Committee of the Erasmus MC (MEC-2-12-176) with patient consent given (or informed consent by parents or guardians in case of children). Human sera were also collected at the Semmelweis University as approved specifically for this study by the ethical committee of Semmelweis University. All animal experiments were approved by Stockholm's Norra djurförsöksetiska nämnd and were conducted in agreement with the European Communities Council Directive 86/609/EEC and the Swedish animal protection legislation. Mice were scored and sacrificed according to the obtained ethical permission.

### Bacterial strains and growth conditions

*M. catarrhalis* strain RH4 was originally isolated from the blood of an infected patient [20] and strain BBH18 was from the sputum of a COPD patient during an exacerbation [21]. Both strains were obtained from Arne Forsgren and Kristian Riesbeck (Malmö University Hospital, Sweden). Bacteria were grown in brain heart infusion (BHI) broth at 37°C with shaking (180 rpm) or on Columbia agar supplemented with 5% sheep blood (Biomérieux, Austria) or horse blood at 37°C.

Additional *M. catarrhalis* strains and clinical isolates (strains from various clinical samples (ear, sinus, nasal cavity and middle ear punctuate samples) were obtained from the Pediatric department at Semmelweis University (Budapest, Hungary), Erasmus University Medical Center (Rotterdam, The Netherlands) or were commercially acquired from GR Micro (London, UK). The following strains (Origin and IDs listed) from GR Micro isolated from patients with acute otitis media were used for the gene conservation studies: Australia (1090122, 1090127, 1091216), Belgium (1510233), Brazil (1041218, 3041116, 3041117), Canada (1022133, 1023257, 2022135), France (1502130, 3502122, 3502129), Germany (3517132, 3518116, 3519121, 3522120), Hungary (3650122, 3650134), Italy (1530120, 2530126, 3530121), Japan (2084130, 2085119, 3079119), Portugal (2560117), South Africa (3681122), South Korea (1070122, 1071124, 2070120), Spain (2554135, 3552130, 3553117), Sweden (3590123, 3590127, 3590135), Taiwan (3696117, 3696119, 3696126), Turkey (2660116, 2660119, 2660122), United States (1001118, 1001207, 1009124, 1009125).

*E. coli* cells were grown in LB broth at 37°C with shaking or on LB plates containing appropriate antibiotics (kanamycin and/or ampicillin).

For human sera adsorption, *E. coli* (DH5α transformed with pHIE11/pMAL9.1 [22]) cells were grown to exponential phase and induced with 0.1 mM IPTG. The bacteria were harvested after one hour and washed three times with ice cold 2x PBS. Prior to addition to serum samples, the pellet was re-suspended in PBS (100 μL per 100 mL culture).

### Selection of human sera for library screening

A comprehensive collection of serum samples was obtained from the Department of Pediatrics, Semmelweis University (Budapest), the Erasmus University Medical Center (Rotterdam), and from Intercell AG (Vienna, Austria). In addition to the sera from otitis media patients, sera from healthy individuals or from patients in other disease groups (asthmatic patients, allergy) were also included in the studies, serving as relevant controls. All sera were aliquoted and stored at −80°C prior to use.

For the preparation of serum pools, all human sera were analyzed by ELISA and Western blot with *M. catarrhalis* cell lysates. The sera containing high titer of antibodies and showing a diversity of bands in the Western blot were selected to create serum pools of 5 individual sera per pool.

### ELISA on bacterial cell lysates and recombinant proteins

*M. catarrhalis* cells were grown in liquid medium at 37°C, 5% CO_2_ until a late log phase was reached. Cells were harvested by centrifugation (1,000 g, 10 min, 4°C) and washed twice with PBS. Bacteria were re-suspended in PBS containing protease inhibitors and then lysed on ice by sonication (2 min, pulse 5, 100% power) and the supernatant was collected by centrifugation. ELISA plates (96F Cert, Maxisorb Nunc-Immuno plate, 439454, Denmark) were coated with either bacterial lysates or recombinant proteins and human serum samples were tested at 3-fold dilutions from 1∶50 to 1∶36,450. Highly specific Horse Radish Peroxidase (HRP)-conjugated goat anti-human IgG (Southern Biotech, 2040-05, USA) was used for signal detection.

### Peptide ELISA

N-terminally biotin-labeled peptides were coated onto streptavidin ELISA plates (Nunc, Denmark) at 5 µg/mL (in a 100 µL volume) and incubated overnight at 4°C. Sera were tested in duplicate at a 1∶1000 dilution. Horse Radish Peroxidase (HRP)-conjugated anti-human IgG antibodies (Southern Biotech) were used according to the manufacturer's recommendation (1∶1000 dilution). ABTS was used as a substrate for HRP and the absorbance read at 405 nm.

### Preparation of IgGs from human serum pools

Prior to library screening, human serum pools were adsorbed against *E. coli* (DH5α transformed with pHIE11/pMAL9.1]) cells in order to reduce background. The cell suspension was added to the serum pools (150 µL cell suspension per 800 µL serum) and rotated overnight at 4°C. The next day, the mixture was centrifuged and the supernatants were transferred into a clean tube. The whole procedure was carried out three times for each single serum pool.

The *E. coli* adsorbed human sera were heat-inactivated at 56°C for 45 min and centrifuged to remove precipitated proteins. The supernatant was filtered using a 0.22 μm syringe filter (Costar, USA) and IgGs were purified and biotinylated with the reagents provided by Pierce Biotechnology (USA), as previously described [17], and subsequently used for library screening.

### Construction of bacterial surface display libraries

Bacterial surface display libraries were generated as previously described [17], [22]. Briefly, genomic DNA from *M. catarrhalis* BBH18 was fragmented by DNase I digest (LamB library, DNase Shotgun Cleavage Kit (Novagen, USA)) or sonication (FhuA library, Sonopuls Ultrasonic Homogenizer HD2200 (Bandelin, Germany)). Blunt-ended DNA fragments of 50–200 bp or 150–600 bp were ligated with the *Sma*I digested frame-selection vector pMAL4.31. pMAL4.31 containing 50–150 bp or 150–600 bp DNA fragments from *M. catarrhalis* was transformed into DH10B electrocompetent *E. coli* cells (Invitrogen, USA). Plasmid DNA was isolated from the pool of transformed clones, and the DNA inserts cloned into the platform vectors pMAL9.1 (FhuA, 150–600 bp) and pHIE14 (LamB, 50–150 bp) for surface display.

### MACS screening

MACS (magnetic-activated cell sorting) screening using bacterial surface display libraries was performed as described previously [17], [22].

### Cloning, expression and purification of recombinant *M. catarrhalis* proteins in *E. coli*

For recombinant expression of *M. catarrhalis* antigens, the PCR amplified gene or gene fragments to be expressed were cloned into pET28b+, a vector containing a kanamycin resistance cassette as well as a T7-RNA polymerase promoter. All proteins were expressed with N- or C-terminal His-tags without possible signal peptides. Protein expression was analyzed in small scale (2 mL) cultures, and protein solubility was determined based on centrifugation of lysed bacterial cultures and analysis of soluble (supernatant) and insoluble (pellet) fractions. Western blot with anti-His-tag antibodies was performed to confirm the expression of the recombinant protein. Proteins were purified from 2 L *E. coli* BL21(DE3) cultures carrying the pET28b+ vector encoding the antigens. Soluble proteins were purified using an IMAC column according to standard methods, insoluble proteins were purified by washing the inclusion bodies, solubilizing them in a buffer containing 6 M Guanidine hydrochloride (GuHCl), and subsequently applying them to an IMAC column. Bound proteins were eluted with 250 mM imidazole in denaturing buffer. Proteins were refolded by dilution with a buffer without GuHCl but containing L-Arginine (0.5 M) as an inhibitor of protein aggregation. After renaturation, proteins were dialyzed against 50 mM Tris-HCl, 150 mM NaCl buffer at pH 8.0 and decreasing concentration of L-Arginine (100 mM final). Alternatively, inclusion bodies were solubilized with 8 M urea and purified under denaturing conditions in the presence of 0.2% N-lauroylsarcosine. Proteins were then dialyzed against PBS, 0.2% N-lauroylsarcosine.

### Preparation of whole cell membranes from *M. catarrhalis*

Cells from a 1.5 L culture were harvested (4,500 rpm, 4°C, 60 min) and washed with PBS. The pellet was re-suspended in 100 mM Na_2_CO_3_ and sonicated on ice for 2 min (50%). After centrifugation (12,000 rpm, 4°C, 10 min) to remove cell debris, the supernatant was ultracentrifuged (40,000 rpm, 4°C, 90 min) and the pellet was washed with PBS (40,000 rpm, 4°C, 90 min). Finally, the pellet was re-suspended in 500 µL PBS.

### Preparation of outer membrane vesicles from *M. catarrhalis*

Cells from a 1.5 L culture (without or with 2 µM Desferal) were harvested (4,500 rpm, 4°C, 60 min) and washed with PBS. The cells were re-suspended in 50 mL EDTA buffer (0.05 M Na_2_HPO_4_, 0.15 M NaCl, 0.01 M EDTA, pH 7.4) and incubated at 56°C for 30 min at 75 rpm agitation with glass beads (1.7–2 mm). The culture was centrifuged (3,500 rpm, room temperature, 15 min) twice, and the supernatant containing the membrane vesicles was ultracentrifuged (40,000 rpm, 4°C, 90 min). The pellet was washed with PBS (40,000 rpm, 4°C, 60 min) and re-suspended in 500 µL PBS.

### Generation of mouse immune serum against *M. catarrhalis* recombinant protein Msp22

Msp22 with a His-tag at the C-terminus and expressed without lipidation in *E. coli* was purified using IMAC columns and utilized for the generation of Msp22-specific immune serum in mice. Female NMRI mice 6–8 weeks of age were bled by tail vein puncture to generate pre-immune sera, and were immunized three times intraperitoneally with 50 µg recombinant antigen per immunization, using Complete Freunds Adjuvant (CFA) or Incomplete Freunds Adjuvant (IFA) as adjuvant. Terminal bleeds were collected via the orbital sinus. Sera were heat-inactivated at 56°C for 30 minutes.

### Immunization and challenge of mice

Five to seven-week-old female C57/BL6 mice were kept under specific pathogen-free conditions in a standardized 12 hours light/dark cycle and received commercial food and water *ad libitum*. Before immunization on Day 0, 10 µL of blood was withdrawn from each mouse to prepare pre-immune serum samples. On days 0, 21 and 42, intranasal immunization of groups of 10 mice as controls with PBS or Intercell's proprietary adjuvant IC31^®^ [23] and with the respective adjuvanted proteins was performed as follows: 17.5 µL protein solution was mixed with 2.5 µL IC31^®^ (2000 nmol/mL KLK: 80 nmol/mL ODN1a), incubated for 30 minutes at room temperature and used to immunize mice within one hour of preparation. Adjuvant control mice received 17.5 µL 50 mM Tris/HCl pH 8.0 mixed with 2.5 µL IC31^®^. Immune sera were obtained on Day 63 (3 weeks after the last boost) and frozen at −20°C for storage.

Twenty-one days after the last boost, mice were infected intranasally with 40 µL (20 µL per nostril) live *M. catarrhalis* strain RH4, equaling approximately 5×10^6^ CFU. For mouse inoculation, *M. catarrhalis* RH4 was grown in BHI broth to an OD_620_ of 0.4. Bacteria were pelleted and re-suspended in PBS. Mice were held in a head-up vertical position during the inoculation and kept in that position for at least 10 seconds after the inoculation.

### Euthanasia, tissue collection and bacterial culture

Mice were euthanized at 6 hours post-infection. Both lungs were removed, placed in 1 mL PBS plus protease inhibitor (Roche, Germany), homogenized using cell strainers (100 µm, Becton Dickinson and Company, USA) and used for serial plating to quantify viable bacteria.

For the evaluation of bacterial clearance due to immunization with recombinant proteins, several independent experiments were performed and the CFU in the lungs of the mice were normalized to an infectious dose of approximately 5×10^6^ CFU bacteria (actual dose varied between 3.8× to 5.9×10^6^ CFU) and analyzed with non-parametric Kruskal-Wallis tests and Dunńs post-testing.

### Preparation of *M. catarrhalis* lysates

*M. catarrhalis* RH4 or BBH18 lysates were prepared from cultures grown in BHI broth. The cells were harvested, washed and re-suspended in PBS, then sonicated on ice using 2×30 second bursts. The protein concentration was measured using BCA protein assay reagent (Pierce Biotechnology).

### Generation of the *msp22* gene deletion mutant

The *M. catarrhalis* gene deletion mutant *msp22*Δ was generated by amplifying a ∼500 bp region up- and downstream of the msp22 gene from genomic DNA using the following oligonucleotide primers: 8666–5′-TGATATTCGCTGAGATGTGA-3′; 8667–5′-CCACTAGTTCTAGAGCGGCAGTGTGGTTCTTGCCATAAG-3′; 8668–5′-GCGTCAATTCGAGGGGTATCTAAAACATGCAGCAGCTAAG-3′; 8669–5′-GATGGCATCATACCAATCTT-3′. The flanking regions of the gene were ligated by overlap-extension PCR with a spectinomycin resistance cassette that was derived from the vector pR412T7 [24]. *M. catarrhalis* cells were rendered competent by washing with PBS containing 0.15% bovine gelatin. Transformation was achieved by adding the DNA fragments to the competent cell cultures, and subsequent plating on spectinomycin-containing blood agar plates (100 µg/mL). The numbers of CFUs were counted after overnight incubation at 37°C. *Msp22* gene-specific PCRs, sequencing and Southern blot analysis were performed to confirm the presence of the gene deletion.

### Msp22 cloning for complementation, expression and purification in *M. catarrhalis* BBH18 using complementation plasmid pEMCJH04-KAN

The complete *msp22* gene and a region of approximately 200 bp upstream of the gene was amplified using genomic DNA as template and primers 8825 and 8826, and cloned into pEMCJH04-KAN [25] resulting in pEMCJH04-KAN-Msp22. Mini prep DNA of pEMCJH04-KAN-Msp22 and primers 8825 and 8860 (primer containing 6xHis-tag) were used for PCR amplification (see listing below). The resulting fragment was *Bam*HI/*Pst*I digested and ligated with *Bam*HI/*Pst*I digested pEMCJH04-KAN (→pEMCJH04-KAN-Msp22-HIS). Transformation of the ligation into competent *M. catarrhalis* wild type and gene deletion mutant *msp22*Δ cells was performed as described above. Transformed cells were plated on blood agar containing 50 µg/mL kanamycin. Clones were analyzed by colony PCR using the following primers: 8825–5′-ATATATGGATCCCATAACATAAATTGCCGTTGTCTTGG-3′; 8826–5′-ATATATCTGCAGCTATTTTTTCTTATAAGCCTTATGGC-3′; 8835–5′-ACTTTTGCTGAGTTGAAGGA-3′, 8836–5′-ACAAAATGTTGTAGCGGTCT-3′; 8860–5′-AAAACTGCAGCTAGTGGTGGTGGTGGTGGTGTTTTTTCTTATAAGC-3′.

### Purification of Msp22 from *M. catarrhalis*

*M. catarrhalis* wild type (negative control) and *M. catarrhalis* cells containing pEMCJH04-KAN-Msp22-HIS were plated on blood agar plates containing 0 or 50 µg/mL kanamycin. Fifteen mL of BHI medium was inoculated with several colonies from the plate and bacteria were grown for 5 hrs (37°C, 180 rpm). The culture was transferred to 150 ml BHI medium and grown overnight. The following day, the cultures were diluted in 1.5 L BHI medium and grown at 37°C, 180 rpm for 5 hrs (wt, negative control) or overnight (*M. catarrhalis* containing pEMCJH04-KAN-Msp22-HIS). The cells were harvested by centrifugation and frozen at −20°C until use.

The pellet was thawed and re-suspended in lysis buffer (50 mM Tris/HCl pH 8.0, 500 mM NaCl, 0.1% Triton X-100) containing protease inhibitors. Sonication on ice was performed 7×2 min (5×10% cycle, 100% power), and soluble and insoluble fractions were separated by centrifugation. Small scale Western blot analysis of crude lysate, soluble and insoluble fractions was performed to determine the solubility of the protein. The protein in the soluble fraction was purified using an IMAC affinity column. The protein bound to the column was washed with Tris/NaCl buffer (50 mM Tris/HCl pH 8.0, 500 mM NaCl, 0.5 mM DTT) containing 0.1% Triton X-100 (wash 0), buffer only (wash 1), 20 mM imidazole (wash 2) and 40 mM imidazole (wash 3) and then eluted in 50 mM Tris/HCl pH 8.0, 150 mM NaCl, 250 mM imidazole.

### Luminol based heme staining

For heme staining, the protocol by Feissner *et al.* [26] was used. Briefly, SDS-PAGE was performed using non-reducing loading buffer and samples were not heat treated prior to loading. Proteins were subsequently blotted onto a nitrocellulose membrane, washed with PBS and incubated with the substrate luminol (SuperSignal West Femto Maximum Sensitivity Substrate Trial Kit, Pierce Biotechnology). Luminol/Enhancer solution and Stable Peroxidase Buffer were mixed at a 1∶1 ratio and added to the membrane, followed by exposure of the membrane to light-sensitive film, allowing the detection of proteins with heme-dependent peroxidase activity.

## Results

### Human sera for antigen identification recognize *M. catarrhalis* proteins

Antigen identification using human sera relies on the assumption that candidate antigens have induced seroconversion or an immune response in patients recovering from infection or in healthy individuals upon encounter with the pathogen without developing disease. For identification of *M. catarrhalis* vaccine candidate antigens, 414 sera from patients (children 1–10 years of age) with otitis media were collected over a three year period. This serum collection included 147 serum pairs taken from the same individual during acute and convalescent disease phase (294 samples) and 120 single serum samples taken either from the acute or convalescent phase from different patients. Human sera were further collected from children suffering from respiratory allergies or asthma (2–18 years) and healthy adults (18–40 years) having no recent history of middle ear disease or *M. catarrhalis* infection. The sera containing high titer of antibodies as measured by ELISA and showing a diversity of bands in the Western blot using whole *Moraxella* cell lysate were selected to create four different serum pools for antigen selection by bacterial surface display (Table 1). In general, good antibody levels against *Moraxella* lysates were detected in the majority of sera, but we could not observe significant differences in IgG levels between samples obtained from patients in acute and convalescent phase. The sera for pooling were therefore selected mainly based on ELISA titer and Western blotting. Sera from both, healthy individuals and patients, had higher ELISA titers than the sera from patients with recurrent AOM, while the latter showed a more homogeneous banding pattern in Western blot as compared to the individual sera included in the other pools. Serum pool PMc36 contained sera from young patients (2–18 years) with respiratory allergies, PMc37 serum pool was derived from children with asthma (5–17 years), PMc39 serum pool included sera from the patients with recurrent otitis media and the serum source for IC20 serum pool were healthy individuals.

**Table 1:** Human sera selected for antigen identification by peptide library screening.

Serum pool	Individual sera	Source	Purpose
**P39**	P4060.2, P4070.2, P4072.2, P4101.2, P4115.2	Patients with otitis media; age: 1-10 years	Antigen selection
**IC20**	IC58B, IC84B, IC85B, IC86B, IC89B	Healthy individuals; age: 18–40 years	Antigen selection
**P36**	P3792, P3801, P3819, P3832, P3861	Patients with respiratory allergies; age: 2–18 years	Control; other condition
**P37**	P3918, P3923, P3941, P3943, P3965	Patients with asthma; age: 5–17 years	Control; other condition

Pool P39 consisted of individual sera collected from OM patients during the convalescent disease phase. Pool IC20 contained sera from healthy individuals. The additional 2 pools were used as controls for otitis media-unrelated antigen reactivity: Pool P37 (patients with asthma, age: 5–17 years). Pool P36 (patients with respiratory allergies, 8 months– 18 years of age).

### Selection of 23 *M. catarrhalis* vaccine candidate antigens by the ANTIGENome technology

In order to apply the ANTIGENome technology for the identification of novel *M. catarrhalis* vaccine candidates, genomic libraries were generated consisting of *E. coli* cells displaying random peptides of *M. catarrhalis* via the FhuA and LamB platforms on the bacterial cell surface. Approximately 600 clones of each library were sequenced in order to determine the quality of the libraries and to calculate the average insert sizes. Average insert sizes of 39 bp (LamB/1), 87 bp (LamB/2) and 199 bp (FhuA) covering the entire *M. catarrhalis* BBH18 genome 33 times (LamB/1), 56 times (LamB/2) and 38 times (FhuA), were represented by a total number of 1.6×10^6^ (LamB/1), 1.2×10^6^ (LamB/2) and 3.6×10^5^ (FhuA) *E. coli* clones, respectively. The first LamB library contained DNA inserts of an average size of 39 bp, therefore a second LamB library was generated with a larger average insert size.

Screening of the three genomic libraries was performed using IgGs purified from the four serum pools, resulting in 13 individual bacterial surface display screens (3 LamB screen with the LamB/1 library, 4 LamB screens with the LamB/2 library, and 6 FhuA screens) to identify novel vaccine antigens. Approximately 800 clones per screen were sequenced and the results matched to annotated ORFs using BLAST searching (http://blast.ncbi.nlm.nih.gov/Blast.cgi). A problem that occurred in the initial screens was the frequent selection of the Hag/MID, UspA1/UspA2H antigens. Therefore, serum pools IC20 and PMc39 were additionally adsorbed against 3 UspA2H and 4 (IC20 IgG pool) or 6 (P39 IgG pool) Hag/MID library clones that covered the immunodominant regions of these proteins. The selection of 6 Hag/MID library clones for adsorption resulted in a strong reduction of Hag/MID clones in the screen using P39 serum pool, and a relative increase in the selection of the remaining antigens.

In total, 214 candidates were selected by the ANTIGENome approach and positively confirmed by Western blot analysis using the human IgG pools that were initially used for library screening. The most frequently selected antigens in all screens included the previously published antigens Hag/MID (493 hits [27]), the UspA1 and UspA2H proteins (131 hits [10]) as well as LbpB (39 hits [28], [29]) and CopB (35 hits [30], [31]). However, a number of less well characterized proteins, such as a TonB dependent receptor (MCR_0076, 13 hits), an outer membrane protein (MCR_1742, 24 hits), a carboxypeptidase (MCR_1010, 48 hits), and MhuA (MCR_0739, 15 hits) were frequently detected in addition to these well characterized antigens. Certain antigens were preferably selected when screening the FhuA library. These candidates included among others: Hag/MID (FhuA: 349 hits vs. LamB: 144 hits); UspA2H (FhuA: 81 hits vs. LamB: 4 hits); UspA1 (FhuA: 39 hits vs. LamB: 7 hits); and the aconitate hydratase (FhuA: 42 hits vs. LamB: 1 hit). In contrast, McmA was found 29 times in LamB screens, but was only selected once using the FhuA library. Many other antigens were identified equally frequent in both screens. These results confirm that the ANTIGENome technology is a very valuable and comprehensive approach for the identification of novel antigens as potential vaccine candidates. Moreover, the utilization of two different surface display libraries, expressing smaller (LamB) and larger (FhuA) peptides, may also – besides mainly linear epitopes – allow for the selection of conformational epitopes.

Following initial antigen identification, several *in vitro* and *in vivo* analyses were performed to further reduce the number of selected vaccine candidates. Initially, all 214 candidates were tested for their gene distribution among 47 *M. catarrhalis* isolates. Based on this PCR analysis, 196 antigens were present in at least 43 of 47 *Moraxella* strains, whereas only 18 candidates were present in less than 90% of all isolates tested.

In order to evaluate the immunogenicity of individual antigens in humans, an ELISA using synthetic peptides corresponding to the epitope bearing regions of the antigenic proteins identified by the genomic screens was performed using the individual sera from the four human serum pools. The peptides were designed based on bioinformatic analysis of the selected clones encoding immunogenic epitopes and synthesized with an N-terminal biotin-tag. In case of longer antigenic fragments (more than 26 amino acid residues), overlapping peptides were generated. The 402 peptides were selected from 110 antigens according to their frequency of being selected by the antigen screens as well as their annotation (e.g. predicted to be surface located, antigenic or secreted peptides/proteins). The 50 most reactive peptides are listed in Tables 2 and 3. Several of the most reactive peptides corresponded to antigens frequently found in the screens, such as PcnB/MCR_1836, GroES/MCR_1494, PrfC/MCR_1681, GidA/MCR_1350, RpoC/MCR_0258, AcnB/MCR_0394 and McmA/MCR_1652.

**Table 2:** ELISA data for the 50 most reactive *M. catarrhalis* peptides – Average ELISA titers for groups of sera.

Peptide	Annotation	Average (OM)	Average (Asthma)	Average (Healthy)	Average (All)
MCR_1292-02	phosphatidylethanolamine Kdo2-lipid A phosphoethanolamine transferase	553	486	507	518
MCR_0412-03	hypothetical protein	413	396	412	407
MCR_1728-03	Ppx/GppA phosphatase	445	410	294	387
MCR_1387-01	ribonuclease PH	428	474	219	377
MCR_1836-07	poly(A) polymerase	479	358	260	373
MCR_0169-04	excinuclease ABC subunit A	511	394	154	363
MCR_1494-02	chaperonin protein Cpn10	431	390	239	359
MCR_0081-02	prolyl endopeptidase	348	377	317	347
MCR_1728-05	Ppx/GppA phosphatase	347	351	276	326
MCR_1596-01	phospholipid/glycerol acyltransferase	333	265	332	311
MCR_1690-04	extracellular solute-binding protein family 3	280	401	241	306
MCR_0036-01	glutamate-cysteine ligase	334	353	211	302
MCR_0604-04	Fe-S protein assembly chaperone HscA	316	291	244	286
MCR_1619-10	ribonuclease E	303	347	178	277
MCR_1200-01	2-isopropylmalate synthase	370	244	173	269
MCR_0036-03	glutamate-cysteine ligase	233	240	338	268
MCR_1283-01	glycine dehydrogenase	340	287	153	265
MCR_0092-01	3-ketoacyl-CoA thiolase FadA	301	260	211	260
MCR_1683-02	DNA polymerase I	304	298	140	251
MCR_1681-01	peptide chain release factor 3	280	231	216	245
MCR_1596-02	phospholipid/glycerol acyltransferase	230	271	230	243
MCR_1487-01	ubiquinone biosynthesis hydroxylase	268	283	158	238
MCR_0131-02	nitric oxide reductase NorB	250	251	144	217
MCR_1320-02	cbb3-type cytochrome c oxidase subunit CcoP	243	273	111	211
MCR_0321-03	lysophospholipase-like protein	217	201	181	201
MCR_0604-02	Fe-S protein assembly chaperone HscA	271	165	152	201
MCR_0131-04	nitric oxide reductase NorB	263	247	69	197
MCR_0996-04	hypothetical protein	248	225	107	197
MCR_0934-05	polyphosphate kinase 2	257	204	108	194
MCR_1003-02	LysM domain protein	169	300	114	193
MCR_1735-02	M48 family zinc metallopeptidase	213	231	129	192
MCR_1295-02	leucyl-tRNA synthetase	222	208	119	186
MCR_0439-03	penicillin-binding protein 1A	194	200	154	184
MCR_0078-01	hypothetical protein	193	211	136	180
MCR_0169-03	excinuclease ABC subunit A	240	226	58	179
MCR_1672-02	pepSY-associated membrane protein	149	229	163	178
MCR_0692-03	hypothetical protein	216	213	96	177
MCR_0092-02	3-ketoacyl-CoA thiolase FadA	276	153	78	176
MCR_0258-01	DNA-directed RNA polymerase subunit beta'	170	156	194	173
MCR_0321-04	lysophospholipase-like protein	191	180	132	169
MCR_0791-02	nicotinate-nucleotide diphosphorylase	184	205	113	168
MCR_0625-01	penicillin-binding protein 1B	210	177	106	167
MCR_0394-04	aconitase	200	93	201	167
MCR_0136-02	conserved hypothetical protein	178	191	129	166
MCR_1690-01	extracellular solute-binding protein family 3	153	207	136	164
MCR_1652-02	peptidase M16 inactive domain protein McmA	183	206	92	162
MCR_1350-06	tRNA uridine 5-carboxymethylaminomethyl modification enzyme GidA	221	178	63	158
MCR_0405-01	tetratricopeptide repeat family protein	145	202	129	158
MCR_1295-01	leucyl-tRNA synthetase	184	134	129	151
MCR_0405-03	tetratricopeptide repeat family protein	184	139	123	151

The peptides are named by the ORF followed by a number indicating the individual peptide for the respective ORF. Individual sera were obtained from asthma patients and healthy individuals and convalescent sera patients with otitis media (OM). Listed are the 50 peptides with highest average ELISA units of the 402 peptides analyzed. ELISA units were calculated as 1,000×[(A_405_ wells with serum) – (A_405_ wells with secondary antibody alone)]. The serum ELISA units were additionally corrected for the background reactivity of sera with streptavidin, by subtracting the values obtained with streptavidin coated wells in the absence of peptide from the values obtained in the wells containing bound peptides.

**Table 3:** ELISA data for the 50 most reactive *M. catarrhalis* peptides – ELISA titers for individual sera.

	OM						Asthma					Healthy individuals					
Peptide	P406 0.2	P407 0.2	P407 2.2	P410 1.2	P411 5.2	P412 0.2	P39 18	P39 23	P39 41	P39 43	P39 65	IC5 8B	IC8 5B	IC8 6B	IC8 9B	IC5 4A	Aver age
MCR_1292-02	677	388	370	789	566	526	706	696	8	626	396	629	504	155	403	846	518
MCR_0412-03	602	68	226	778	411	390	420	416	423	505	216	339	518	0	446	755	407
MCR_1728-03	612	352	197	753	393	365	391	428	424	381	427	253	311	0	356	552	387
MCR_1387-01	506	397	172	672	448	373	404	383	1008	392	185	192	139	0	64	700	377
MCR_1836-07	569	624	433	484	349	414	252	500	393	399	244	247	386	0	233	435	373
MCR_0169-04	472	1008	159	574	408	444	336	635	355	531	111	152	134	64	64	357	363
MCR_1494-02	462	408	196	705	407	409	376	428	658	321	169	189	232	0	136	640	359
MCR_0081-02	522	83	199	623	398	261	464	471	456	478	18	277	420	0	271	617	347
MCR_1728-05	520	58	180	668	364	294	428	426	391	399	109	195	416	0	208	560	326
MCR_1596-01	236	306	117	619	311	408	258	366	257	253	191	318	217	0	721	402	311
MCR_1690-04	139	248	120	663	210	299	300	350	417	692	247	102	524	65	57	455	306
MCR_0036-01	353	215	117	631	334	354	372	346	506	305	237	201	204	0	122	530	302
MCR_0604-04	334	398	303	354	178	331	442	459	292	260	0	203	121	0	562	335	286
MCR_1619-10	396	0	83	671	330	336	450	357	458	209	259	198	94	0	80	516	277
MCR_1200-01	398	784	103	502	237	194	341	273	297	230	78	141	104	0	165	455	269
MCR_0036-03	277	106	275	114	147	477	429	92	253	150	277	214	386	0	715	375	268
MCR_1283-01	346	338	115	602	355	286	397	353	312	258	115	97	88	0	131	450	265
MCR_0092-01	187	822	76	273	182	264	132	475	102	379	212	253	201	93	253	255	260
MCR_1683-02	458	9	121	517	454	264	257	266	604	281	83	102	269	0	48	283	251
MCR_1681-01	284	725	109	205	153	205	167	388	124	291	183	161	706	0	95	119	245
MCR_1596-02	180	271	98	363	153	313	199	208	214	394	338	727	140	0	96	188	243
MCR_1487-01	158	132	84	567	318	351	412	470	0	243	288	86	115	0	78	512	238
MCR_0131-02	173	468	141	300	211	207	211	327	181	208	327	168	129	115	103	204	217
MCR_1320-02	158	112	60	538	308	284	288	354	255	194	274	46	86	0	55	367	211
MCR_0321-03	214	126	0	471	238	255	257	222	26	210	292	247	89	27	157	383	201
MCR_0604-02	186	346	161	370	261	301	191	162	155	224	94	122	123	0	245	272	201
MCR_0131-04	31	945	79	77	85	361	93	580	25	272	265	78	73	0	86	106	197
MCR_0996-04	352	260	11	521	154	192	277	284	350	173	39	55	54	80	0	345	197
MCR_0934-05	232	376	48	449	182	256	254	301	258	193	15	42	68	0	29	400	194
MCR_1003-02	124	233	0	432	40	182	727	216	199	242	118	73	76	0	143	280	193
MCR_1735-02	122	202	35	474	215	229	238	298	207	167	244	74	88	0	84	399	192
MCR_1295-02	223	65	35	593	228	189	282	295	210	254	0	69	67	0	58	400	186
MCR_0439-03	95	312	103	299	180	173	144	159	242	173	284	374	132	0	70	196	184
MCR_0078-01	177	0	132	462	177	207	319	284	252	198	0	73	97	0	0	508	180
MCR_0169-03	176	576	59	359	74	193	404	259	100	322	45	30	10	0	17	233	179
MCR_1672-02	247	290	78	168	100	10	240	101	410	112	282	123	99	143	60	390	178
MCR_0692-03	348	229	56	341	163	158	205	266	256	148	189	95	70	0	48	265	177
MCR_0092-02	325	313	226	97	427	265	103	67	201	89	307	117	73	0	69	131	176
MCR_0258-01	157	143	34	468	102	114	193	240	132	216	0	52	0	0	666	253	173
MCR_0321-04	199	119	0	341	191	296	252	192	19	202	235	168	66	0	94	332	169
MCR_0791-02	285	61	80	308	216	152	218	214	153	233	206	39	159	0	78	287	168
MCR_0625-01	202	139	53	476	204	187	226	272	200	188	0	35	46	0	46	405	167
MCR_0394-04	297	513	76	44	202	65	78	51	98	81	159	490	2	0	305	208	167
MCR_0136-02	83	154	58	368	200	202	237	278	170	166	103	150	65	0	70	359	166
MCR_1690-01	162	136	91	306	112	108	205	165	350	152	164	172	157	0	56	294	164
MCR_1652-02	109	119	4	474	159	234	297	278	241	158	57	57	11	0	13	380	162
MCR_1350-06	245	317	143	307	165	147	193	242	145	126	185	1	10	0	0	306	158
MCR_0405-01	128	202	45	237	150	106	179	229	239	116	249	43	42	292	35	232	158
MCR_1295-01	158	167	71	224	216	265	164	122	109	169	104	131	117	0	70	329	151
MCR_0405-03	534	106	46	232	154	29	214	129	118	101	132	168	154	0	70	225	151

For legend see Table 2.

A final selection of 23 promising antigens for recombinant protein production and further *in vivo* evaluation was made based on the number of screen hits, data obtained from the serological studies, and the bioinformatic and gene distribution analyses (see Table 4). All 23 candidates were present in at least 44 of the 47 tested *Moraxella* isolates and the majority of the antigens were predicted to be localized in the outer membrane. In addition, proteomic studies with *M. catarrhalis* membrane fractions were performed to support antigen selection (data not shown). As shown in Table 4, four candidates (OppA, M16-like peptidase, MhuA and MsrAB) were found in all membrane preparations, whole membrane, outer membrane vesicles and outer membrane vesicles isolated from cultures grown in iron-depleted medium (as a variety of virulence factors are induced by low iron levels). Three candidates (hypothetical proteins MCR_0063, MCR_0691, MCR_0692) were found in the whole membrane and in outer membrane vesicles, and seven further candidates were detected in one of the three membrane preparations.

**Table 4:** *M. catarrhalis* antigens selected by the ANTIGENome technology.

ID	Annotation	aa	GD	Hits	1	2	3	4	5
MCR_0063	hypothetical protein	232	47/47	8	+	+		PP (9.84)	−
MCR_0076*	TonB-dependent receptor	913	44/47	13				OM (9.52)	−
MCR_0136	hypothetical protein	278	47/47	2				PP (9.84)	+
MCR_0186	outer membrane lipoprotein LolB	190	46/47	6	+			? (2)	−
MCR_0196*	MltB; lytic murein transglycosylase	473	47/47	12	+			IM (9.97)	+
MCR_0439	Pbp1A; penicillin-binding protein 1A	786	47/47	5				IM (9.82)	+
MCR_0560	hypothetical protein	355	44/47	5				IM (10)	−
MCR_0681	putative lytic transglycosylase	303	45/47	7				? (5.02)	+
MCR_0686*	peptide methionine sulfoxide reductase MsrA/MsrB	558	47/47	3	+	+	+	CP (9.26)	+
MCR_0691	hypothetical protein	105	46/47	4	+	+		? (2.5)	+
MCR_0692	hypothetical protein	503	45/47	7	+	+		? (2.5)	+
MCR_0739	hemoglobin utilization protein MhuA	954	46/47	15	+	+	+	OM (10)	+
MCR_0918	M16-like peptidase	470	46/47	5	+	+	+	? (2)	+
MCR_0996*	hypothetical protein	146	47/47	3		+		PP (9.84)	+
MCR_1003*	LysM domain protein	819	47/47	9	+			OM (9.49)	+
MCR_1010*	DacC; D-alanyl-D-alanine carboxypeptidase	386	47/47	48				PP (9.76)	−
MCR_1228	D15 surface antigen family protein	907	47/47	4	+			OM (9.52)	−
MCR_1303*	OppA; oligopeptide ABC transport system substrate binding protein	679	47/47	6	+	+	+	? (5.02)	+
MCR_1357	Cyt1; cytochrome c1 family protein	241	47/47	22				? (2.5)	−
MCR_1416*	cytochrome c class II Msp22	152	47/47	2				PP (9.44)	+
MCR_1690	extracellular solute-binding protein family 3	262	44/47	6		+		PP (10)	+
MCR_1742	outer membrane protein	111	46/47	24				? (2.5)	+
MCR_1761	OlpA; OPA-like protein A	235	47/47	7	+			OM (10)	−

aa, amino acids; GD, gene distribution; 1, Proteins detected in the whole membrane preparation; 2, Proteins detected in outer membrane vesicles (iron-rich conditions); 3, Proteins detected in outer membrane vesicles (iron-depleted conditions); 4, Bioinformatic analysis, predicted localization using PSORTb3.0.3 (score), OM  =  outer membrane, PP  =  periplasmic, IM  =  inner membrane, CP  =  cytoplasmic, ?  =  unknown; 5, Peptide ELISA (+; at least one peptide with an average ELISA unit ≥100); *selected for *in vivo* studies.

### Three candidate vaccine antigens demonstrated protection *in vivo*

**Figure 1:**
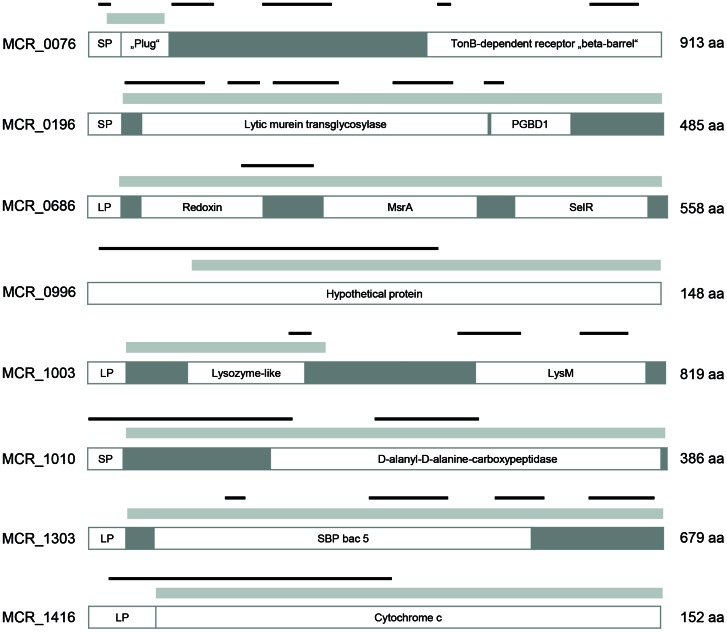
Structural features of 8 potential *M. catarrhalis* vaccine candidates. MCR_0076, TonB-dependent receptor; MCR_0196, MltB; lytic murein transglycosylase; MCR_0686, peptide methionine sulfoxide reductase MsrA/MsrB; MCR_0996, hypothetical protein; MCR_1003, LysM domain protein; MCR_1010, D-alanyl-D-alanine carboxypeptidase; MCR_1303, oligopeptide ABC transport system substrate binding protein; MCR_1416, cytochrome c class II, Msp22. SP, signal peptide; LP, signal peptide for lipidation; Plug, an independent folding subunit blocking the pore until the channel is bound by a ligand; PGBD1, peptidoglycan binding-like; MsrA, methionine sulfoxide reductase A; SelR, seleno protein R; LysM, lysine motif; SBP bac 5, bacterial extracellular solute-binding protein family 5. Light grey bars represent the recombinant protein (fragments). Thin black bars delineate epitope containing regions covered by clones selected by the ANTIGENome technology with human IgGs.

**Figure 2:**
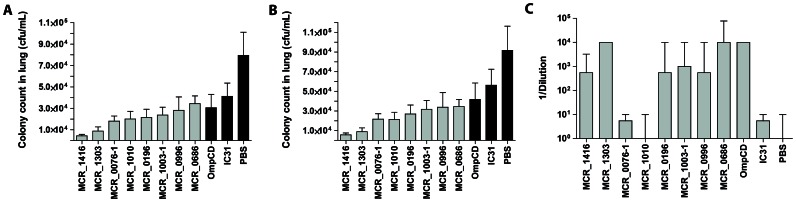
Pulmonary clearance of *M. catarrhalis* RH4 after intranasal challenge following intranasal immunization with 8 selected antigens. Pulmonary clearance 6 hours after intranasal challenge with ∼5×10^6^ CFU *M. catarrhalis*, in mice immunized with purified, IC31^®^ adjuvanted recombinant proteins, IC31^®^ adjuvant without proteins in PBS, or PBS without adjuvant. The mean values of the combined, normalized results from 2 to 6 independent experiments are shown. Error bars represent the standard error of the mean. (A) Bacterial CFU recovered from all experiments; (B) bacterial CFU recovered from experiments after exclusion of sterile lung cultures. Black bars: negative and positive controls (data from 6 experiments), grey bars: data from 2 to 3 independent experiments in which different antigens were tested. (C) ELISA measuring IgG levels to the respective recombinant proteins in serum from mice immunized intranasally with purified recombinant proteins as noted below the x-axis. For the controls (IC31^®^ alone or PBS), IgG levels were determined using a mix of all recombinant proteins. Endpoint titers were expressed as the last dilution that gave an absorbance of at least 0.1 at 405 nm. Median values with the interquartile range from 2 to 6 independent experiments using 10 sera (10 mice per group) per experiment are shown. **, statistically highly significant (P<0.01), *, statistically significant (P<0.05).

Of the 23 candidates selected by the ANTIGENome technology, we evaluated 8 well conserved (see Table 5) and readily recombinant expressed antigens that had shown some promise in a preliminary mouse study in more detail for their potential to elicit protective immune response *in vivo* (Figure 1). The rate of *M. catarrhalis* clearance from mouse lungs in response to immunization with recombinant antigens was assessed using a mouse pulmonary clearance model (Figure 2). Mice were immunized intranasally 3 times at 3 week intervals and challenged intranasally with 40 µL of approximately 5×10^6^ live *M. catarrhalis* RH4 (actual CFU varied between 3.8×10^6^ to 5.9×10^6^) 3 weeks after the last boost. Bacterial CFU were determined in lungs 6 hours post infection and systemic antibody titers after vaccination of mice were determined by ELISA (Figure 2).

**Table 5:** Properties of aligned polypeptide sequences for 8 potential *M. catarrhalis* vaccine candidates.

ORF	aa Start-Stop	Length	No. of non-synonymous/deleted aa	No. of isolates
MCR_0076	21–160	140	10	62
MCR_0196	36–485	450	32	63
MCR_0686	28–558	531	28	64
MCR_0996	27–148	122	21	64
MCR_1003	30–375	346	7#	64
MCR_1010	27–386	360	21	64
MCR_1303	24–679	656	31	64
MCR_1416	21–152	132	6	64

Sequences were aligned using the Bionumerics algorithm (Bionumerics v 6.0 software, Applied Maths) and default settings. Length, length in translated amino acids. #, a single insertion event of 12 amino acids was also observed in a single isolate for this vaccine candidate.

Groups of mice immunized with recombinant proteins MCR_1416, MCR_1303, MCR_0076-1, MCR_1010, MCR_0196, MCR_1003-1, MCR_0996 and MCR_0686 expressed in *E. coli* showed a greater or comparable clearance of bacteria from lungs compared to the positive control protein OmpCD (Figure 2A, B). The effect was statistically significant for MCR_1416 with one log reduction in bacterial recovery compared to mice immunized with adjuvant alone (IC31^®^) (p<0.01) (Figure 2A). Further, there was also a significant reduction in bacterial load for MCR_1303 (p<0.05) and MCR_0076-1 (p<0.05) compared to IC31^®^ alone, when sterile lung cultures were removed from the analysis (Figure 2B). The exclusion of sterile cultures was considered reasonable, based on the observation that negative (sterile) lung cultures appeared randomly between 0 to 3 in the 6 PBS groups, the number of sterile lung cultures in the immunized mice occurred with the same frequency as in the PBS groups (between 0 and 4). Therefore, the sterile lung cultures were more likely to represent a technical artifact (infection failure), rather than elimination of bacteria. While significant protection was observed for MCR_1416, MCR_1303 and MCR_0076-1, protection was lower for the other candidates despite strong antibody responses as measured by IgG ELISA (Figure 2C). In contrast, the IgG response was very low for MCR_0076-1 and MCR_1010, while the level of protection was higher than for the positive control protein OmpCD. This observation indicated that factors other than antibody responses may contribute to protection against *M. catarrhalis*.

### Systemic human antibody responses against the selected antigens are not induced upon infection

In order to evaluate the human immune response for the 8 selected recombinant antigens upon natural infection, additional serological studies were performed with ELISA and Luminex xMAP^®^ technology, using a collection of 164 individual sera from children with otitis media collected during the acute and convalescent disease phase. Sera from healthy individuals were tested in parallel in order to compare antigen specific responses between healthy adults and children with otitis media. We detected antibodies against all eight antigens in the 20 paired acute/convalescent serum samples from children with otitis media, however IgG end titers were relatively low (<2000) and no significant antigen specific seroconversion (defined as ≥2 fold increase in the convalescent IgG titer) was detected in any of the donors (data not shown). We also examined the median antibody titers between healthy donors and otitis media patients, however no statistically significant difference was seen (data not shown). Moreover, we detected a decrease in median systemic IgG titers against the antigen MCR_1303 in convalescent sera compared to acute sera (data not shown). These results are in agreement with the peptide ELISA data, as no increase in antibody titer was detected for these antigens in sera from otitis media patients during an OM episode when the paired serum samples were collected.

### MCR_1416 exhibits heme-dependent peroxidase activity

The recombinant antigen showing the highest protection in the pulmonary clearance model was further studied for its biological function. MCR_1416 has previously been identified as *Moraxella* surface protein 22 (Msp22) [9] and shows homology to cytochrome *c*, it containing one CXXCH motif (residues 142 to 146). C-type cytochromes are characterized by covalent attachment of heme to the protein via two thioether bonds formed between the heme vinyl groups and the cysteine sulfurs in a CXXCH peptide motif [32]. Since Msp22 also contains this motif, we set out to determine whether it binds heme and exhibits heme-dependent peroxidase activity. Heme staining was performed according to the method of Feissner *et al.* [26] using luminol as substrate for the heme-dependent peroxidase activity.

**Figure 3:**
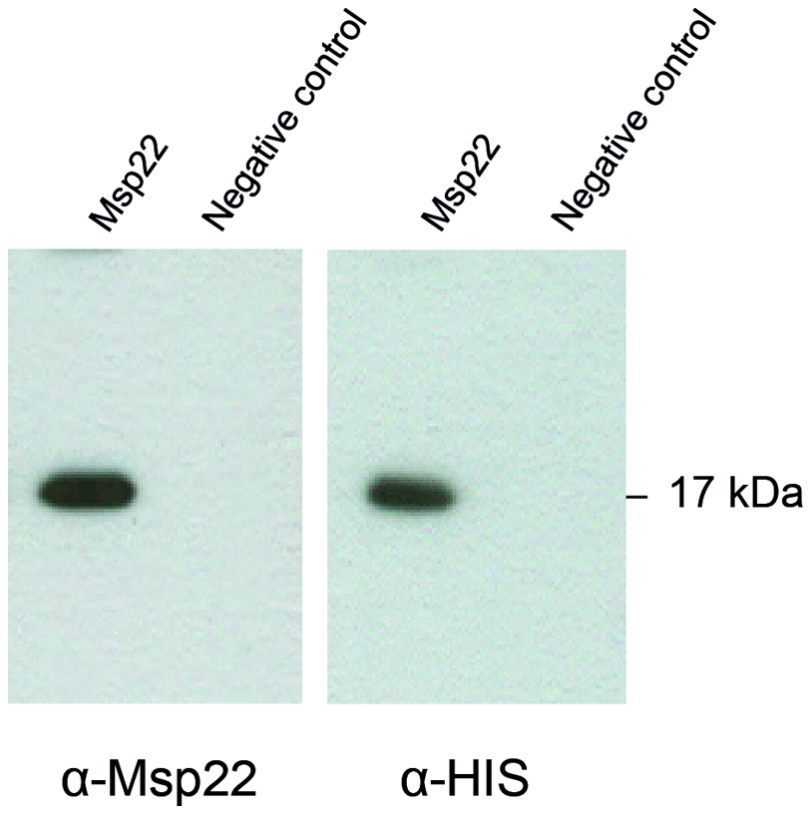
Detection of recombinant MCR_1416 (Msp22) expressed and purified from *M. catarrhalis*. Equal volumes of eluates obtained from IMAC columns from extracts of *M. catarrhalis* complemented with His-tagged MCR_1416 (eluate A) or wild type strain (not complemented, negative control) were separated by SDS-PAGE and immunoblotted using immune serum against recombinant Msp22 (left panel) and antibody against the His-tag (right panel).

In order to try to ensure that native lipidated Msp22 protein was recovered possessing its correct native conformational folding, Msp22 with its native signal sequence and a C-terminal His-tag was expressed in *M. catarrhalis* (i.e. its native host), using the complementation vector pEMCJH04-KAN. We complemented the wild type strain with the plasmid expressed Msp22 in order to increase the yield of purification from *M. catarrhalis*. Subsequently, Msp22 was obtained from the soluble fraction and purified on an IMAC column. Western blot analyses of the column eluate (using extracts from *M. catarrhalis* with or without pEMCJH04-KAN-Msp22-HIS) and immune sera against recombinant MCR_1416 and anti-penta-His antibody revealed successful purification of His-tagged Msp22 (Figure 3). These experiments also showed that the Msp22 protein as produced by wild type *M. catarrhalis* is recognized by antibodies induced in mice by the recombinant *E. coli* protein.

**Figure 4:**
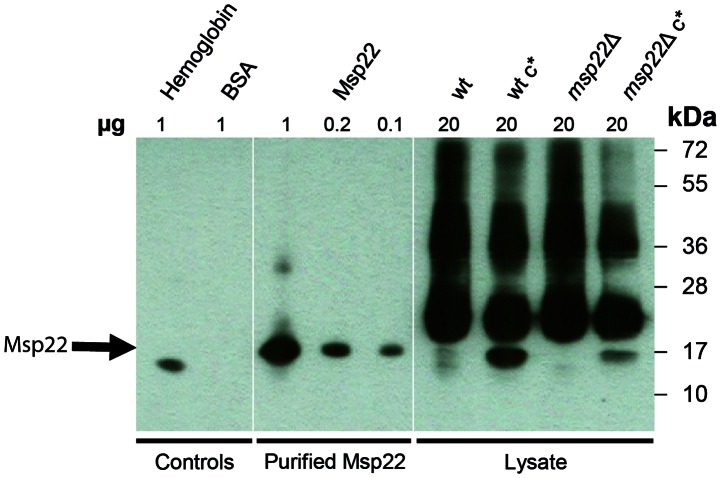
Msp22 shows heme-dependent peroxidase activity. The specificity of the heme stain for Msp22 is demonstrated by staining of lysates from the wild type, and gene deletion mutant strains as well as the BBH18 strain transformed with pEMCJH04-KAN-Msp22. Hemoglobin (positive control), BSA (negative control). wt, wild type *M. catarrhalis* BBH18; wt c*, wild type *M. catarrhalis* BBH18 transformed with pEMCJH04-KAN-Msp22; msp22Δ, msp22 gene deletion mutant; msp22Δ c* msp22 gene deletion mutant transformed with pEMCJH04-KAN-Msp22. The position of Msp22 is marked with an arrow.

For heme detection experiments, samples were prepared using a non-reducing sample buffer and were not heated prior to SDS-PAGE, which was performed under denaturing conditions. Bacterial lysates of the wild type, the *msp22* gene deletion mutant and the complemented strains all served as additional heme controls. Positive signals were obtained for hemoglobin, purified Msp22 and the cell lysates expressing Msp22, indicating the presence of heme-dependent peroxidase activity. No signal was detected for the negative control protein BSA (Figure 4). The absence of the respective protein band at 17 kDa in the *msp22* gene deletion mutant, the presence of a strong signal in the complemented strain, and a weak signal in the wild type strain suggested that the 17 kDa hemoprotein was indeed Msp22.

## Discussion

Over the last three decades, *M. catarrhalis* has become recognized as an important pathogen of the human respiratory tract [33], [34], [35], [36], [37], [38], [39]. However, even though *M. catarrhalis* is the third most frequent bacterial pathogen to be associated with otitis media and is a major cause of exacerbations of COPD in adults, none of the currently available bacterial vaccines developed to prevent these diseases include *M. catarrhalis* antigens [8]. Therefore, the aim of this study was to comprehensively identify potential vaccine targets of *M. catarrhalis* by applying the ANTIGENome technology that had previously been developed by Intercell AG (Vienna, Austria), and which had been previously successfully used for vaccine discovery for several other bacterial pathogens [17], [18], [40]. Genomic libraries displaying multiple epitopes of all potential antigens of isolate BBH18 were screened using human IgG pools from patients as well as healthy individuals, anticipating identification of antigens expressed during infection *in vivo*. The most frequently selected antigens in the screens were Hag/MID and UspA1, which are vaccine candidate antigens previously identified in other studies. Hag/MID has been described as an adhesin, a hemagglutinin, and a stimulator of B cells [41], [42], whilst UspA1 functions as an adhesin and a transporter [43], [44]. Further, immunization with UspA1 has been shown to induce bactericidal antibodies in mice and humans [11]. In fact, the detection of these well-known candidate antigens shows the value of the ANTIGENome technology in identifying potential vaccine candidates, also including potentially novel vaccine candidates. Indeed, using this technology, allowed the identification of 214 antigenic *M. catarrhalis* proteins, with 23 of these candidates being further evaluated in a murine *M. catarrhalis* pulmonary clearance model.

The fact that *M. catarrhalis* is a strictly human pathogen, which does not induce active infection in animals, means that there is currently no clinically relevant model for *M. catarrhalis* vaccination studies available, especially for studies that adequately mimic otitis media infection in humans. For this reason, the mouse pulmonary clearance model is the most frequently used animal model to test the ability of antigens to generate a protective immune response against *M. catarrhalis* [45]. However, it is known that mice do not develop pneumonia and are able to clear the *M. catarrhalis* bacteria relatively quickly in this model (within 6–24 hours), and in this study, *M. catarrhalis* clearance occurred within 24 hrs post-infection. It is precisely for this reason that the clearance of *M. catarrhalis* from the respiratory tract was measured at 6 to 9 hrs post-infection when using this animal model, rather than measuring total bacterial clearance at 24 hrs (by which time non-vaccination related factors could have influenced the clearance of the *M. catarrhalis* bacteria) [46]. Based on our preliminary studies with heat killed bacteria and the OmpCD antigen, the optimal end point for *M. catarrhalis* strain RH4 in our model was 6 hrs post-infection. This model was found to be reproducible, as we detected similar clearance rates for the tested antigens in up to 6 independent experiments. Nevertheless, it should be noted that the clearance rate of bacteria from the lungs of vaccinated mice (a measure of the efficacy of vaccination) was based on an actual increase in clearance rate compared to the normal clearance rate observed in unvaccinated control mice. In our study, this meant that the maximum clearance rate we observed using this model lay in the range of 0.5 to 1.0 log10 when compared to negative controls. However, our results are in agreement with similar studies that have previously been performed using putative *M. catarrhalis* vaccine antigen candidates [47], [48], [49].

Using our comprehensive screening technology, we eventually selected 8 out of the 23 proteins that possessed the potential to become vaccine candidates for testing in a mouse pulmonary clearance model. Three of these protein antigens showed beneficial effects on bacterial clearance from mouse lungs after mucosal immunization: 1) MCR_1416 (Msp22), a candidate also previously identified by Ruckdeschel and colleagues [9], [49]; 2) MCR_1303 (OppA), an oligopeptide permease A [50] and 3) MCR_0076, the “plug” domain of a TonB-dependent receptor. The fact that similar results and clearance rates were obtained independently by other investigators for Msp22 [49] and OppA [50] using different experimental set-ups, indicates that these proteins are indeed promising vaccine candidates. MCR_0076, the plug domain of TonB-dependent receptor, is situated within the beta-barrel structure and appears to be more conserved than the barrel. This plug domain is an independent folding subunit blocking the pore until the channel is bound by a ligand and causes the structural and functional differences between these transporters and porins [51], [52], [53]. TonB-dependent receptors have previously been reported to be potential vaccine antigens and important virulence factors [54], [55], [56] and should thus be taken into consideration and analyzed in more detail for *M. catarrhalis*. The *oppA* gene (MCR_1303) encodes an oligopeptide permease that belongs to the ABC transport system. These types of transporters have been shown to play a role in virulence, to be immunogenic and to be potential vaccine candidates [57]. The Msp22 antigen (MCR_1416) induced the most significant *in vivo* protection and was analyzed *in vitro* in more detail in order to explore its function. Due to its homology to cytochrome c and the presence of a CXXCH motif, known to be involved in heme binding, we tested whether this antigen was indeed a heme binding protein. Our heme staining experiment (Figure 4) demonstrated that heme had indeed been covalently attached to the highly soluble Msp22 protein, indicating that Msp22 may exert its function via heme binding.

The heme group of type *c* cytochromes accepts electrons from the bc1 complex and transfers them to the cytochrome oxidase complex. Among other functions, cytochrome *c* has heme-dependent peroxidase activity and plays a role in initiation of apoptosis in more complex organisms [58], [59], [60], [61]. Based on its homology to cytochrome c and its heme binding, Msp22 may also function in the electron transfer via its heme-dependent peroxidase activity. Besides its important role for cytochrome function, heme is also the most abundant source of iron in the human body [62]. Not surprisingly, due to very limited free iron availability in the human host, many pathogens have evolved mechanisms to utilize heme containing proteins as iron sources. Recently, two *M. catarrhalis* proteins have been shown to acquire iron from hemin and heme complexes [63], [64]. Therefore, Msp22 could also be involved in iron acquisition from heme and heme-containing compounds. Interestingly, it was recently suggested that Msp22 has a potential role in divalent ion transport [50]. An investigation into the mechanism of heme binding and the contribution of the CXXCH motif was recently performed for two putative cytochrome *c* peroxidases of *Campylobacter jejuni* [26], [65]. While these proteins exhibited heme binding, site-directed mutations within the CXXCH motif resulted in unstable proteins excluding them from further analysis [65]. Whether this holds true also for *M. catarrhalis* Msp22 remains to be elucidated.

As targets for protective immune responses need to be accessible on the bacterial surface and knowing that Msp22 has been annotated as a putative surface protein, we attempted to confirm the cell surface location of Msp22. However, using flow cytometry of both wild type and Msp22 overexpressing strains and polyclonal anti-Msp22 mouse sera, we could not detect this protein on the bacterial surface. This suggests that the protein is not surface exposed under the *in vitro* growth conditions tested in these studies. In order to elicit a protective immune response, one may speculate that Msp22 may become transiently exposed to the host's immune system during infection. Unlike Msp22, OppA is accessible on the bacterial surface *in vitro* [50] as confirmed by our studies (data not shown), and antibody mediated neutralization of bacteria is therefore likely to be an important protective immune mechanism complementing native immune defenses against this antigen.

Interestingly, in agreement with the data obtained by other researchers in this field [50], we could not detect significant differences in the antibody titers against the 8 tested antigens in; 1) sera from children with otitis media in the acute compared to the convalescent disease phase, or 2) in sera from children compared to sera from healthy individuals. The natural systemic IgG response observed in humans has therefore not provided any further validation of our selected eight antigens, but the selection as a vaccine candidate was rather based on the pulmonary clearance model. Furthermore, although UspA1, UspA2 and Hag/MID antigen specific antibodies were frequently found in both children and healthy individuals [66], [67], there is no clear evidence that natural immune responses raised against other putative vaccine candidates contribute to protection. The question whether naturally induced antibodies against any *M. catarrhalis* antigens play a role in protection against otitis media has been previously raised [50], and our observations confirm that further investigations into the immune mechanisms operating during *M. catarrhalis* infection induced by this pathogen will be required. In addition, naturally occurring antibodies may exhibit different epitope specificity and avidity, compared to vaccine induced antibodies. But more importantly, systemic IgG levels do not adequately reflect mucosal immune responses. Thus, if mucosal immunity is more critical for protection against *M. catarrhalis*, serological studies based on serum samples collected from otitis media patients may be of limited value. Such a discrepancy between mucosal and systemic serological immune responses was previously detected in otitis media patients against *M. catarrhalis* outer membrane antigens [68]. In addition, the role of T cells for protection and B cell activity stimulation remains to be elucidated. Most recent studies suggested that *M. catarrhalis* is able to modulate mucosal epithelial responses and B cell adaptive immunity in such a way as to hinder the generation of antibodies with a correct function and epitope specificity [69], [70]. If this indeed turns out to be the case, vaccination with *M. catarrhalis* would be an extremely valuable approach in preventing infection by this pathogen. In terms of antigen validation, the detection of a natural immune response against the selected antigens indicated that they were expressed *in vivo* upon infection of the human host.

In conclusion, comprehensive screening using the ANTIGENome technology has led to the identification of 214 antigenic proteins, with 3 of these being shown to provide protection against *M. catarrhalis* colonization in a mouse pulmonary model. The results confirm that further evaluation of these proteins as vaccine candidates in additional functional studies and in clinically relevant *Moraxella* otitis media models is warranted.
